# A Comparative Analysis of Industrial *Escherichia coli* K–12 and B Strains in High-Glucose Batch Cultivations on Process-, Transcriptome- and Proteome Level

**DOI:** 10.1371/journal.pone.0070516

**Published:** 2013-08-08

**Authors:** Karoline Marisch, Karl Bayer, Theresa Scharl, Juergen Mairhofer, Peter M. Krempl, Karin Hummel, Ebrahim Razzazi-Fazeli, Gerald Striedner

**Affiliations:** 1 Department of Biotechnology, University of Natural Resources and Life Sciences, Vienna, Austria; 2 Austrian Centre of Industrial Biotechnology, Vienna, Austria; 3 Institute for Genomics and Bioinformatics, Graz University of Technology, Graz, Austria; 4 VetCORE Facility for Research, University of Veterinary Medicine, Vienna, Austria; Indian Institute of Science, India

## Abstract

*Escherichia coli* K–12 and B strains are among the most frequently used bacterial hosts for production of recombinant proteins on an industrial scale. To improve existing processes and to accelerate bioprocess development, we performed a detailed host analysis. We investigated the different behaviors of the *E. coli* production strains BL21, RV308, and HMS174 in response to high-glucose concentrations. Tightly controlled cultivations were conducted under defined environmental conditions for the in-depth analysis of physiological behavior. In addition to acquisition of standard process parameters, we also used DNA microarray analysis and differential gel electrophoresis (Ettan^TM^ DIGE). Batch cultivations showed different yields of the distinct strains for cell dry mass and growth rate, which were highest for BL21. In addition, production of acetate, triggered by excess glucose supply, was much higher for the K–12 strains compared to the B strain. Analysis of transcriptome data showed significant alteration in 347 of 3882 genes common among all three hosts. These differentially expressed genes included, for example, those involved in transport, iron acquisition, and motility. The investigation of proteome patterns additionally revealed a high number of differentially expressed proteins among the investigated hosts. The subsequently selected 38 spots included proteins involved in transport and motility. The results of this comprehensive analysis delivered a full genomic picture of the three investigated strains. Differentially expressed groups for targeted host modification were identified like glucose transport or iron acquisition, enabling potential optimization of strains to improve yield and process quality. Dissimilar growth profiles of the strains confirm different genotypes. Furthermore, distinct transcriptome patterns support differential regulation at the genome level. The identified proteins showed high agreement with the transcriptome data and suggest similar regulation within a host at both levels for the identified groups. Such host attributes need to be considered in future process design and operation.

## Introduction

*Escherichia coli* is the most used microorganism in biological research laboratories and the biotech industry because of its fast growth and the achievement of high cell densities in inexpensive media [1]–[3]. Various microbial expression systems are applied for recombinant protein production, whereby a great number of plasmids or genome-integrated host/vector combinations are available [4], [5]. Different cultivation conditions are used with a great emphasis on the achievement of high product quality and yield [6], [7]. Nevertheless, the interaction of the target protein with cell metabolism remains little understood, which is an obstacle to efficient process design. Consequently, a profound knowledge of the cellular host applied is very important for rapid establishment of new recombinant protein production processes and optimization of established processes. Growth characteristics of a specific host can provide limited systems information. For a more detailed understanding of individual properties, monitoring techniques must involve enhancement at the molecular level. Techniques that are useful include microarray analysis or real-time polymerase chain reaction (PCR) for measuring gene expression levels and two-dimensional gel electrophoresis for determination of protein expression.

Northern blot analysis in *E. coli* has revealed higher gene expression levels related to acetate metabolism and glyoxylate shunt in the BL21 compared to the K–12 strain JM109, and an acetate-control mechanism has been proposed [8], [9]. In this context, Shiloach et al. suggested that differential acetate regulation might explain the lower acetate formation during cultivations of BL21 relative to K–12 strains [9]. In addition, other metabolic pathways have exhibited differential regulation, such as the fatty acid pathway or glycolysis as a whole [8]. Furthermore, *E. coli* BL21 can achieve greater amounts of biomass and higher growth rates than *E. coli* K–12 strains, possibly because of differentially regulated glucose transport, as recently suggested [10]. Moreover, in BL21, insertion elements have functionally disabled two proteases (*lon*, *ompT*) [11].

The objective of the current study was to evaluate the performance of frequently used *E. coli* hosts–B strain BL21 and K–12 strains HMS174 and RV308–in the context of industrial cultivations [2], [3], [12]. To meet this objective, we carried out batch cultivations with high-glucose media in computer-controlled bioreactors to ensure constant growth conditions in terms of oxygen supply and pH and applied transcriptome and proteome analysis to identify phenotypic attributes at the molecular level. This is in contrast to a similar comparison study of *E. coli* K–12 and B hosts by Yoon et al. where simple shake flasks were used [13]. In addition, we compared two different K–12 hosts to the well-known BL21 strain, which are mainly industrially applied strains [2].The resulting enhancement of knowledge about host physiology is expected to contribute to the acceleration of improvements and optimizations in related biotech processes.

The experimental set-up allowed for maximum growth levels of each strain; consequently, we could focus on strain-specific features based on differences among the hosts. Additionally, acetate formation is potentially triggered due to glucose-overflow conditions during batch cultivations. For “–omics” analyses, we used DNA microarrays and 2-D fluorescence difference gel electrophoresis (Ettan^TM^ DIGE, also referred to as 2D-DIGE). Transcription analysis was performed on two custom-designed, sequence-specific BL21 and K–12 microarrays [14]. Protein identification was based on peptide mass fingerprints as well as tandem mass spectrometric analyses of selected peptides using a matrix-assisted laser desorption/ionization tandem time-of-flight mass spectrometer. A reference design using an internal standard consisting of a pool of all analyzed samples enabled comparison of the different strains and was used for transcriptome and proteome analysis. The presented approach serves as both, a useful comparison of these strains and a model strategy for performing this type of analysis.

## Materials and Methods

Chemicals were purchased from VWR unless otherwise stated.

### Bacterial strains

The strain *E. coli* RV308 was purchased from the American Type Culture Collection (ATCC # 31608); *E. coli* HMS174 and *E. coli* BL21 from Novagen (Germany). Strains did not contain a plasmid, T7 system (lambda DE3 phage) or any other recombinant protein expression component.

### Cell cultivation

Batch cultivations were carried out in a computer-controlled bioreactor from MBR Wetzikon (CH) with a batch volume of 4 L and a total reactor volume of 20 L. The semi-synthetic media used for all cultivations contained 3 g KH_2_PO_4_ and 6 g K_2_HPO_4_⋅3 H_2_O per liter. These concentrations provided the required buffer capacity and served as P and K sources, as well. The other components were added in relation to the cell dry matter (CDM) (per g) to be produced: 0.25 g C_6_H_5_Na_3_O_7_⋅2 H_2_O, 0.10 g MgSO_4_⋅7 H_2_O, 0.01 g CaCl_2_⋅2 H_2_O, trace element solution 50 μL, 0.45 g (NH_4_)_2_SO_4_, 0.37 g NH_4_Cl, and 3.30 g glucose⋅H_2_O. The complex components yeast extract (0.05 g) and tryptone (0.1 g) were added to the medium to accelerate initial growth of the population.

Trace element solution, prepared in 5 N HCl (per liter) contained: 40.0 g FeSO_4_⋅7 H_2_O, 10.0 g MnSO_4_⋅H_2_O, 10.0 g AlCl_3_⋅6 H_2_O, 4.0 g CoCl_2_, 2.0 g ZnSO_4_⋅7 H_2_O, 2.0 g Na_2_MoO_2_⋅2 H_2_O, and 1.0 g CuCl_2_⋅2H_2_O, and 0.50 g H_3_BO_3_.

Overnight cultures for batch inoculation, prepared in 2000 mL shake flasks containing 320 mL semi-synthetic medium (3 g CDM/L), were grown at 180 rpm and 37°C on an orbital shaker. The bioreactor was inoculated with a calculated amount of ∼0.4 g of CDM for all batch runs, and a final biomass of 53.2 g could be achieved (40 g/L glucose in the media).

The cultivation conditions were kept constant during all batch cultivations. The temperature was controlled at 37±0.5°C, and the pH was maintained at a set point of 7.0±0.05 by addition of 25% ammonium solution (w/w) (Acros Organics). The oxygen level was controlled at 30% saturation by stirrer speed and aeration rate control. Foaming was suppressed by addition of 0.5 mL antifoam suspension (polypropylene glycol, Dow Chemical) per liter. The off-gas analysis (O_2_/CO_2_) was carried out using a Hartmann and Braun Advanced Optima gas analyzer.

### Motility test

Motility was tested by assessing swimming phenotypes on motility agar. Cultures of RV308, HMS174 and BL21 were spotted onto agar plates of LB agar (5 g yeast extract, 10 g NaCl, 10 g tryptone, 3 g agar agar) and semi-synthetic agar (semi-synthetic media containing 3 g agar agar). Plates were incubated at 30°C overnight.

### Sample preparation

#### Sampling for process parameter analysis

Optical density at 600 nm was measured with a spectrophotometer (Amersham Biosciences Ultrospec 500 Pro). CDM was determined by centrifugation of 10 mL of cell suspension. The supernatant was transferred to an Eppendorf vial, frozen at −20°C, and analyzed by high-performance liquid chromatography (1100 HPLC, Agilent Technologies) with an Aminex HPX-87H ion exclusion column (Biorad), 0.01 N H_2_SO_4_ (20°C and 0.45 mL/minute flow rate) as mobile phase with UV/visible-light (Knauer) and refractive index detectors (Beckmann).

The cells were washed with 7 mL distilled water and after resuspension transferred to a pre-weighed beaker. The beaker was dried at 105°C for 24 h and re-weighed. Sample preparation for the determination of nucleotides was performed as described in [15]. The stress-relevant nucleotides, ppGpp and cAMP, as well as their precursors ADP, GTP, and ATP, were separated and quantified by ion-pair reversed-phase HPLC [15].

#### Sampling for subsequent microarray and EttanTM DIGE analysis

For RNA isolation and 2D-DIGE, samples were taken from triplicate runs at the exponential growth phase (Table 1). For RNA analysis, samples were drawn directly into a 5% phenol–ethanol stabilizing solution and split into aliquots corresponding to about 3 mg CDM. Samples for subsequent proteome studies were split into aliquots containing approximately 2 mg CDM. Aliquotes were centrifuged for 2 min at ∼11,000 g at 4°C. The supernatant was discarded, and the pellet was immediately frozen at −80°C.

**Table 1:** Performed cultivations and key process parameters.

Batch run	Host strain	Sampling hour (microarray/ 2D-DIGE) with corresponding CDM (g/L)	Average growth rate (h^−1^)	End biomass CDM (g/L)	Maximum acetate in supernatant (g/L)
HMS1	HMS174	9 (10.02 g/L)	0.47	13.33	6.25
HMS2	HMS174	9 (8.49 g/L)	0.40	14.41	6.00
HMS3	HMS174	9 (9.81 g/L)	0.43	13.43	5.54
RV1	RV308	9 (11.87 g/l)	0.50	15.16	6.01
RV2	RV308	9 (12.38 g/L)	0.47	14.88	4.70
RV3	RV308	9 (11.35 g/L)	0.46	17.49	5.89
BL1	BL21	5.25 (11.07 g/L)	0.73	17.74	1.37
BL2	BL21	5.25 (9.58 g/L)	0.74	15.60*	1.18
BL3	BL21	5.25 (10.32 g/L)	0.73	17.9	1.34

Data shows average growth rates, final cell dry mass (CDM) at the end of cultivations, and maximum achieved acetate concentration in the supernatant for all cultivations. In addition, the sampling times for transcriptome (microarrays) and proteome analyses (Ettan^TM^ DIGE) during the cultivations is given with the corresponding cell dry mass concentration in g/L.

(* Sample was drawn before end of cultivation.)

### Transcriptome analysis

The transcriptome of BL21, RV308 and HMS174 cells at the late exponential growth phase were compared to the pooled reference containing all samples.

#### Microarrays

Microarrays comprising selective probes (70-mer oligonucleotides) for all open reading frames of the *E. coli* K–12 (MG1655) or *E. coli* BL21 genome (operon custom array, Germany). The oligonucleotides were spotted onto an epoxy surface. The mRNA expression level of 3882 unique genes could be measured with both arrays and was consequently considered for the strain comparison (indicated as commonset). The experimental data (including all processing protocols) can be downloaded via ArrayExpress (http://www.ebi.ac.uk/microarray-as/ae/ [16]). The ArrayExpress accession number for the BL21 array design is A-MARS-11 and for the K–12 array design A-MARS-12. The experiment with the K–12 array (HMS174 and RV308) has an ArrayExpress accession number E-MARS-20, and the experiment with the BL21 array has the accession number E-MARS-21.

#### Preparation of RNA: Isolation of RNA

RNA was isolated by TRIzol® reagent (Invitrogen) pulping and chloroform (Sigma-Aldrich, St. Louis, MO, USA) extraction according to the modified protocol described by Hedge et al., 2000 [17]. The quality of RNA was checked on a RNA1000 LabChip® using the Agilent Bioanalyzer according to the protocol of the supplier (RNA integrity number of eight or higher; Agilent Bioanalyzer, Application Guide), and the RNA concentration was determined with the NanoDrop1000.

#### Reverse transcription and indirect labeling

Ten micrograms of total RNA were labeled with a different fluorescent dye (Cy3 or Cy5, GE Healthcare) and then subjected to paired competitive hybridizations. Indirect labeling involves two steps: incorporation of amino-allyl dUTP by reverse transcription and then attachment of the fluorescent dyes.

Briefly, total RNA was mixed with 3 µg of random primer (hexamer) in a total volume of 15.5 µL, denatured at 65°C for 10 min, and primed during cooling prior to RT. To this mixture was added 0.6 µL of 50x dNTP mix [10 mM each of dATP, dGTP, and dCTP; 4 mM of dTTP; and 6 mM amino-allyl dUTP (Sigma-Aldrich, St. Louis, MO, USA)], 6 µL of 5x first-strand buffer, 3 µL 0.1 M DTT (both provided with Superscript III reverse transcriptase), 0.25 µL RNAsin (Promega), and 2 µL of Superscript III reverse transcriptase (Invitrogen). After 2 h incubation at 42°C, 10 µL of 0.5 M EDTA and 10 µL of 1 M sodium hydroxide were added and placed at 65°C for 15 min. After cooling to room temperature, 25 µL of 1 M Tris-HCl (pH 7.5) were added, and cDNA was washed and concentrated using a Microcon-YM30 (Millipore). Finally, cDNA was dried in a speedvac. The dried cDNA was stored at −20°C until use. The cDNA was resuspended in 4.5 µL 0.2 M sodium carbonate buffer (pH 8.5–9.0) and mixed with 4.5 µL of monoreactive Cy3 or Cy5 dye resuspended previously in 37 µL of DMSO. The cDNA–dye mixture was incubated for 1 h at 23°C in the dark. For dye quenching, 4.5 µL of 4 M hydroxylamine was added and incubated again for 15 min at 23°C in the dark. Labeled cDNA was mixed with 35 µL of 3 M sodium acetate (pH 5.2) and purified using a Qiagen MinElute PCR Purification Kit according to the manufacturer's protocol. The incorporation of Cy-dye into the cDNA was measured with the NanoDrop1000, and 200 pg of a Cy3-labeled sample was combined with 200 pg of a Cy5-labeled sample, mixed with 2 µL of salmon sperm (10 mg/mL stock, Invitrogen), and hybridized on a microarray.

#### Array processing

Blocking and hybridization of the microarrays was carried out using the TECAN HS400 hybridization station. The slides were blocked with blocking buffer (4x SSC, 0.5% (v/v) SDS, 1% BSA) for 1 h and then washed with water and 2x SSC containing 0.1% SDS. One sample and the reference pool (containing all samples) were combined in one tube and dried to a volume of ∼7 µL in a speedvac, mixed with OpArray HybSolution (Operon) to achieve a concentration of 90% and a final sample volume of 70 µL, and heated to 95°C for 3 min. The samples were hybridized onto microarrays in dye-swap pairs. Hybridization was carried out for 16 h at 49°C. After hybridization, slides were washed with 1x SSC and finally with 0.5x SSC for 1 min. Slides were dried and scanned using the Agilent™ microarray scanner at 10 µm resolution.

#### Statistical analysis of microarray data

Resulting images were analyzed with Dapple Version 0.88pre2 [18], where the data was extracted. Data analysis was performed using the statistical computing environment R Version 2.15.1 [19].

For standard low level analysis only foreground intensities were considered, i.e., no background correction was performed. The data were preprocessed using print-tip loess normalization. Differential expression estimates (log-fold change) were calculated using R package limma [20].

When searching for candidate's only genes with average log2 intensity (A) larger 7.5, log-fold change (M) larger (±1.0) and corresponding p-value smaller 0.05 at least in one strain were considered. Visualization of gene expression was performed using R package gcExplorer [21].

#### Verification of microarray data – NanoString nCounter gene expression system

RNA was isolated as described above and analyzed using the NanoString nCounter gene expression system, which captures and counts individual mRNA transcripts [22]. Advantages over existing platforms include direct measurement of mRNA expression levels without enzymatic reactions or bias, sensitivity coupled with high multiplex capability, and digital readout. The sensitivity of the NanoString nCounter gene expression system is similar to that of real-time PCR. The transcript levels for selected genes across all samples showed similar patterns of gene expression for microarray data and NanoString data (see FileS1.xlsx).

### Proteome analysis – Ettan^TM^ DIGE

#### 2-D electrophoresis

Protein concentration of sample aliquots was determined with the 2-D Quant Kit (GE Healthcare) and samples were dissolved in cell lysis buffer (8 M urea, 30 mM Tris and 4% CHAPS, pH 8.0 to 9.0) to obtain a final protein concentration of 2.5 µg/µL of lysate and placed on ice for immediate usage.

Samples were labeled following the minimal labeling concept according to the instructions of GE Healthcare. The internal standard was labeled with Cy2 (reference pool consisting of all analyzed samples) and the samples with Cy3 or Cy5. Two samples and the reference were run on the same gel. Two-dimensional electrophoresis was performed as described in [23] with the following modifications: For isoelectric focusing, 300 µg of total protein extract was loaded in triplicate to middle-range immobilized pH gradient (IPG) strips, pH 4–7, 24 cm, and pH 6–11, 18 cm. To detect biological variations of samples derived from the same cultivation, equal amounts of CDM were applied to each IPG strip. Separation in the first was performed on the Ettan™ IPGphor II at 20°C using a voltage gradient according to the recommendation of the manufacturer (GE Healthcare). Homogeneous gels (12.5%T) were prepared for the second dimension using the Ettan™ gel casting chamber. 2D gels were run in the Ettan™ chamber using the EPS 600 Power Supply (GE Healthcare).

#### Optical detection and data evaluation

The Cy-dye labeled gels were scanned with the Typhoon 9400 Variable Mode Imager (GE Healthcare) immediately after the run. The Decyder software package (Version 5.0) was used for data evaluation, and proteins showing an absolute average ratio >2.0 and Student's t-test value of <0.01 were considered to be significantly altered in abundance and subjected to subsequent spot and protein identification by matrix-assisted laser desorption/ionization-mass spectrometry (MALDI-MS). Because of the occurring mass shift [24], gels were post-stained with the ProteoSilver™ Plus Silver Stain Kit according to the manufacturer's instructions (Sigma-Aldrich, St. Louis, MO, USA) before spot picking.

#### Reduction and alkylation

Selected spots were picked manually, and destaining was performed according to the protocol of the ProteoSilver™ Plus Silver Stain Kit. Reduction with dithiothreitol (10 mM) in 100 mM aqueous ammonium bicarbonate (Acros Organics, Geel, Belgium) (60 min shaking on the thermomixer at 56°C) and subsequent alkylation with iodoacetamide (Acros Organics, Geel, Belgium) (55 mM) in 100 mM aqueous ammonium bicarbonate (45 min at room temperature) were performed according to [25]. After being washed with ammonium bicarbonate (100 mM aq.) three times, samples were dried with acetonitrile (Fisher Scientific, Pittsburgh, PA, USA) and vacuum centrifugation.

#### In-gel digestion

In-gel digestion with trypsin (Trypsin Gold, mass spectrometry grade, Promega, Madison, WI, USA) was performed according to the manufacturer and [25]. Lyophilized trypsin powder was reconstituted in 50 mM acetic acid to a concentration of 1 µg/µL. Before use, 5 µL of trypsin stock solution were diluted with 120 µL water, 120 µL ammonium bicarbonate (100 mM aq.), and 10 µL calcium chloride (120 mM aq.) to a final concentration of 20 ng trypsin per µL. Ten µL of trypsin solution were added to each dried spot. After 20 min of swelling, excess trypsin solution was removed, and the spots were covered with ammonium bicarbonate buffer (50 mM) for digestion on the thermomixer at 37°C for 18 h. Afterwards, peptides were extracted with three changes of 30 µL acetonitrile: water:formic acid (50:45:5 v/v) supported by ultrasonication for 15 min per change. Extracted peptides were dried down in a vacuum concentrator.

#### Desalting

To improve ionization of peptides and mass spectrometric sensitivity, dried peptides were re-dissolved in 10 µL 0.1% aqueous trifluoroacetic acid solution and desalted using ZipTips C18 (Millipore, Billerica, MA, USA) according to the Millipore User Guide.

#### Spotting and mass spectrometric analysis

One µL of desalted peptides was spotted onto an AnchorChip MALDI target plate (600 µm anchor size, Bruker Daltonics, Bremen, Germany) and mixed with 2 µL α-cyano-4-hydroxycinnamic acid (0.3 g/L α-cyano-4-hydroxycinnamic acid in ethanol:acetone 2:1). Mass spectrometric data were acquired on a Bruker Ultraflex II matrix-assisted laser desorption/ionization tandem time-of-flight mass spectrometer (Bruker Daltonics, Bremen, Germany) in MS and MS/MS mode. Spectra processing and peak annotation were done using FlexAnalysis and Biotools (both Bruker Daltonics, Bremen, Germany).

For standard database searches, processed spectra were searched via Mascot (Matrix Science, http://www.matrixscience.com/ [26]) in the Swiss-Prot database with the following search parameters: taxonomy: *E. coli*, global modifications: carbamidomethylation of cysteine, variable modifications: oxidation of methionine, MS tolerance: 100 ppm, MS/MS tolerance: 1 Da, one missed cleavage allowed. MS and MS/MS identifications were considered to be statistically significant at a significance level of α<5%.

## Results and Discussion

### Description of datasets

To evaluate *E. coli* strain-specific qualities for host selection, experiments were designed to ensure that the different strains were grown under unlimited nutrient conditions in batch cultivation which triggered sensitivity to acetate accumulation [9]. To characterize overall host attributes, standard process parameters like cell dry matter (CDM) and concentrations of residual glucose and metabolites, such as acetate in the supernatant, were analyzed by high-performance liquid chromatography (HPLC). To detect host-specific reprogramming of the metabolism and strain-specific attributes, samples for transcriptome (microarray) and proteome (Ettan^TM^ DIGE) profiling in the exponential growth phase were additionally assayed. The resulting data delivered in-depth information that will give a complete picture of specific host characteristics and help in future for fine-tuning processes.

### Cultivations

For each host strain, three identical cultivations with a standardized amount of inocula (for details see Materials and methods section) were conducted. Within a specific host experiment, reproducible growth curves were obtained (Table 1). Mean values including standard error of the mean were used to characterize the replicates.

**Figure 1:**
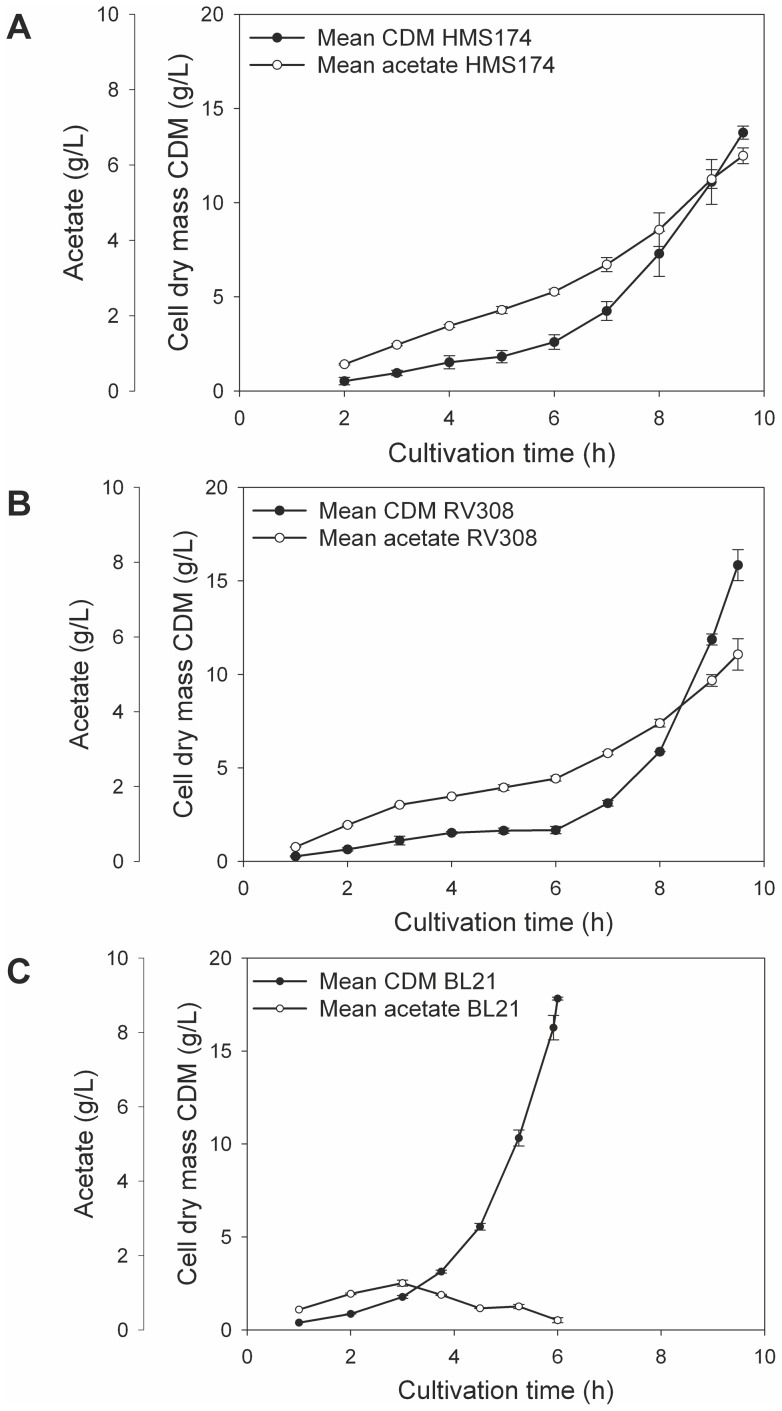
Batch growth and acetate data. Bacterial growth (cell dry mass; CDM) and acetate production in the supernatant of the three replicate cultivations of HMS174 (A), RV308 (B) and BL21 (C) showing the mean values including standard error of the mean.

**Figure 2:**
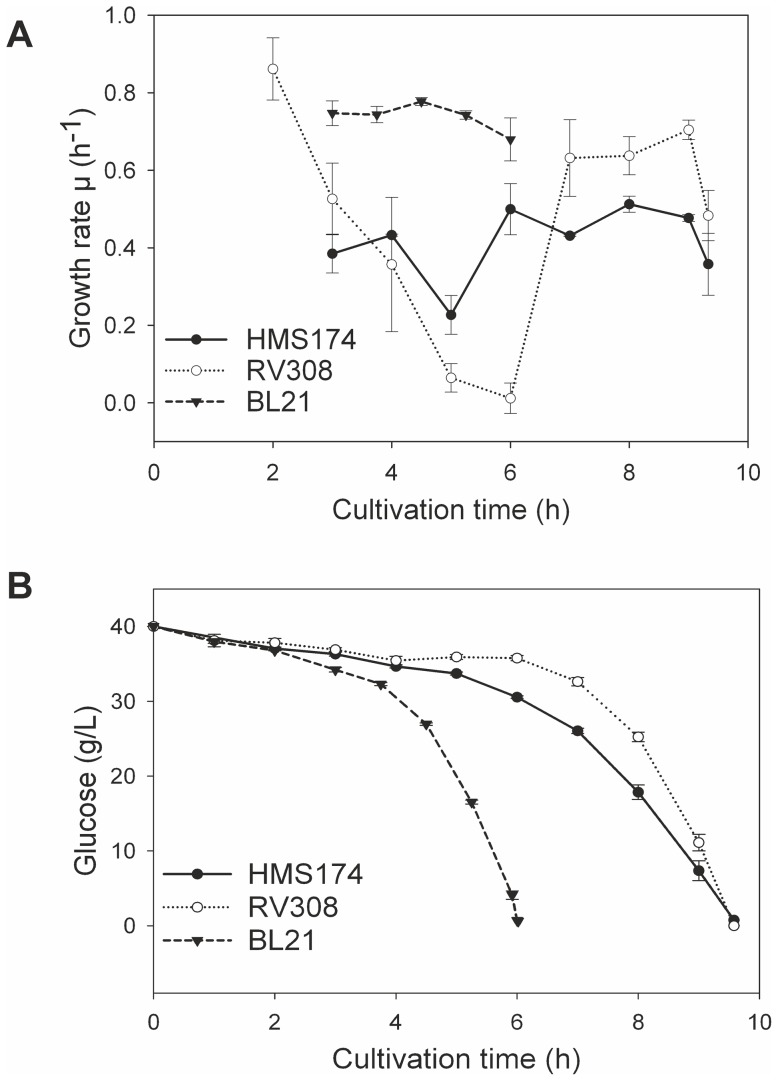
Growth rates and glucose trend. Calculated growth rates of the three *E. coli* hosts (A) and course of glucose consumed by the three different strains (B) during the batch cultivations.

The three cultivations of the *E. coli* K–12 HMS174 reached a final biomass concentration of 13.72±0.34 g/L after 9.6 h (Table 1, Figure 1A). The average growth rate was 0.42±0.03 h^−1^ for all cultivations (Figure 2A). The acetate was accumulated in correlation with the CDM and reached 5.93±0.21 g/L at the end of the exponential growth phase.

The *E. coli* strain RV308 reached a final biomass of 15.84±0.83 g/L during the cultivations (Table 1, Figure 1B). After a prolonged lag phase of 6 h post inoculation, growth rate increased sharply up to 0.70±0.03 h^−1^ on average (Figure 2A) until the glucose was fully consumed after 9.6 h. The average growth rate for the entire batch cultivation was 0.48±0.01 h^−1^, and the course of biomass and acetate accumulation confirmed the high reproducibility of the three cultivations. The maximum amount of acetate in the supernatant reached 5.53±0.42 g/L at the end of the exponential growth phase.

The average growth rate of *E. coli* BL21 was highest at 0.73±0.01 h^−1^ and constant during exponential growth (Figure 2A). The batch cultivations were finished in 6 h compared to about 9.5 h for the K–12 strains (Table 1, Figure 1C). The final biomass reached 17.41±0.11 g/L of CDM on average.

The lag phase of RV308 was not observed in the HMS174 or BL21 experiments. A reprogramming of the metabolism might be the cause for this long adaptation phase after the complex yeast extract in the batch media was presumably consumed (e.g. adaption of amino acid metabolism).

BL21 showed an increase of acetate at the beginning up to about 1.30±0.10 g/L followed by a slight decrease in contrast to the K–12 strains. Possible explanations for the decline in acetate in the supernatant are acetate consumption, conversion to other metabolites, or a generally reduced acetate formation rate. Shiloach et al. [9] proposed a control mechanism of acetate in BL21, suggested to be activated at acetate concentrations of around 1 g/L. The same trend was observed in the current cultivations and corresponding acetate values. The lower acetate accumulation during cultivation might enable the B strain to reach higher growth rates because acetate is known to inhibit cellular growth [27]. A detailed analysis of the acetate-related genes was performed in this study using transcriptome data.

Altogether, the BL21 cultivations achieved the highest biomass yields and growth rates and lowest acetate accumulation compared to the other strains. The K–12 strains HMS174 and RV308 were comparable in terms of average growth rates but differed in a longer lag phase and higher biomass yields for RV308.

### Carbon balances

To elucidate the differences in carbon utilization among the three strains, carbon (C) balances were calculated. The elemental composition of *E. coli* biomass was taken from literature and applied for all three hosts [28].The balances of the batch cultivations confirmed a more efficient use of glucose in terms of biomass accumulation for BL21 and RV308 (Table 2). About 16% of carbon in all K–12 batch cultivations was converted into acetate and were therefore not available for CDM production. The carbon dioxide output was in agreement with the achieved CDM in all hosts. Although, no oxygen limitations were identified during the cultivations, the pO_2_ level was kept constantly above 30%; small amounts of lactate and formate were formed during the later stage of the processes. We concluded that mixed-acid fermentation triggered by the glucose overflow metabolism caused the formation of these metabolites. Additionally, low levels of pyruvate were detected in the supernatant. Around 10% of carbon could not be assigned to any metabolites measured in HMS174 and BL21, but this percentage was in the error range of the applied methods.

**Table 2:** Carbon balance.

	Elemental formula	HMS174		RV308		BL21	
		(g)	(%)	(g)	(%)	(g)	(%)
C in biomass	CH_1.77_O_0.49_N_0.24_	26.36±0.38	45.59	30.43±0.39	52.30	32.81±0.83	59.68
C in acetate	CH_3_COO^−^	9.69±0.18	16.76	9.05±0.11	15.56	0.70±0.14	1.28
C in formate	HCOO^−^	0.19±0.07	0.32	0.28 ±0.18	0.48	0.31±0.07	0.56
C in carbon dioxide	CO_2_	14.35±0.26	24.82	16.79 ±0.01	28.87	16.16±0.16	29.40
C in pyruvate	C_3_H_3_O_3_	0.83±0.13	1.44	0.15±0.01	0.26	non-detectable	
C in lactate	C_3_H_5_O_3_	0.30±0.04	0.52	0.25±0.03	0.43	0.25±0.02	0.46
***C total***			***89.45***		***97.90***		***91.37***

Carbon balances of the three strains calculated at the end of the batch experiments for each strain separately and showing the mean values including the standard deviation of the mean, indicating carbon use for different metabolites produced during cultivation. The elemental formula of *E. coli* was taken from literature [28]. Metabolites were measured in the supernatant (acetate, formate, pyruvate, or lactate) or by off-gas analysis (carbon dioxide).

The C balance (Table 2) fully reflected the differences at the metabolic level and provided fundamental information for host selection but requires closer analysis for other parameters, such as the transcriptional pattern of carbon transport.

The substrate yield coefficients (Y_X/S_) for the three strains also reflected the different growth behavior caused by the distinct substrate exploitation. Figure 1B shows the decrease in glucose consumed by the growing cell populations, which involved a very fast use of glucose for BL21, followed by HMS174 and RV308. In addition, BL21 had the highest biomass per substrate ratio at 0.45±0.00 g/g, followed by RV308 with 0.40±0.04 g/g, and HMS174 with 0.34±0.01 g/g. However, both K–12 strains accumulated approximately 6 g/L of acetate during the cultivations, which also had to be considered in terms of the biomass yield coefficient. Therefore, the 6 g of the K–12 acetate consumed 9 g of glucose, and for the 1.3 g of acetate in the BL21 cultivations, about 2 g of glucose were wasted for by-product accumulation. As a result, the carbon available was reduced to 31 g/L for the K–12 strains and 38 g/L for the B strain, which changed the biomass per substrate ratio to 0.46 g/g for BL21, 0.51 g/g for RV308, and 0.44 g/g for HMS174. Thus, the biomass achievable in a simple batch process was higher for BL21 than for both K–12 strains, but if acetate accumulation can be prevented by an improved process-control or metabolic engineering strategies [29]–[32], RV308 would be the candidate of choice because of the highest biomass per substrate ratio.

### Quantification of metabolic stress

To quantify the metabolic stress level during the cultivations, key analytes of nutrient starvation and cellular stress response, in particular the total amount of cyclic adenosine monophosphate (cAMP) and guanosine 3′-diphosphate 5′-diphosphate (ppGpp), were analyzed by HPLC [15]. cAMP, an important signaling molecule that participates in the regulation of transcription of more than 100 genes, acts through a cAMP receptor protein (CRP) [33], [34] and was detected at higher levels in BL21 batch cultivations (Table 3). cAMP is an indicator for glucose starvation, so the high values for the nucleotide during the batch cultivations are not easily explained because glucose was available. A possible explanation for the phenomenon is an overall higher cAMP level in BL21.

**Table 3:** HPLC analysis of nucleotides.

HMS174	time (h)	cAMP (µmol/g CDM)	ppGpp (µmol/g CDM)
	6.00	0.59±0.41	0.81±0.57
	7.00	0.81±0.00	0.84±0.17
	8.00	0.68±0.03	0.74±0.16
	9.00	0.65±0.06	0.52±0.03

RV308	time (h)	cAMP (µmol/g CDM)	ppGpp (µmol/g CDM)
	6.00	0.83±0.01	1.97±0.31
	7.00	0.75±0.06	0.95±0.08
	8.00	0.57±0.04	0.40±0.00
	9.00	0.39±0.01	0.27±0.05

BL21	time (h)	cAMP (µmol/g CDM)	ppGpp (µmol/g CDM)
	4.50	1.22±0.13	0.86±0.12
	5.25	1.34±0.22	0.55±0.06
	6.00	1.48±0.34	1.70±0.72

Analysis of the nucleotides and stress-relevant markers cAMP and ppGpp (mean values including the standard deviation of the mean) during the batch cultivations. Nucleotides were analyzed by HPLC according to [15].

The small nucleotide ppGpp is the effector molecule of the stringent response in *E. coli* triggered by uncharged transfer RNAs (tRNAs) or C limitation [35]. A higher level of ppGpp was measured in RV308 during the long lag phase. This result indicates an enhanced stress level for this strain and an encountered shortcoming of amino acids. This high level of ppGpp most likely coincided with the depletion of the complex media components of yeast extract. The resulting adaptation of cellular metabolism could target a different amino acid metabolism or defect synthesis pathway in RV308, a possibility requiring further investigation (e.g. genome sequence analysis). Similar amounts of ppGpp were found at the end of the BL21 batch cultivations, indicating a high stress level caused by glucose depletion. Except for the high ppGpp values during the lag phase of RV308, the stress level of this host was not elevated during any cultivation as long as glucose was available.

### Proteome and transcriptome analysis of *E. coli* strains

For closer insights into cellular reactions, we used a combined analysis of transcriptome and proteome techniques, applying microarray analysis and Ettan^TM^ DIGE to samples of the late exponential growth phase with similar cell dry weight of all cultivations (RV308 and HMS174 at h 9, BL21 at h 5.25) (Table 1).

### Transcriptome analysis

**Figure 3:**
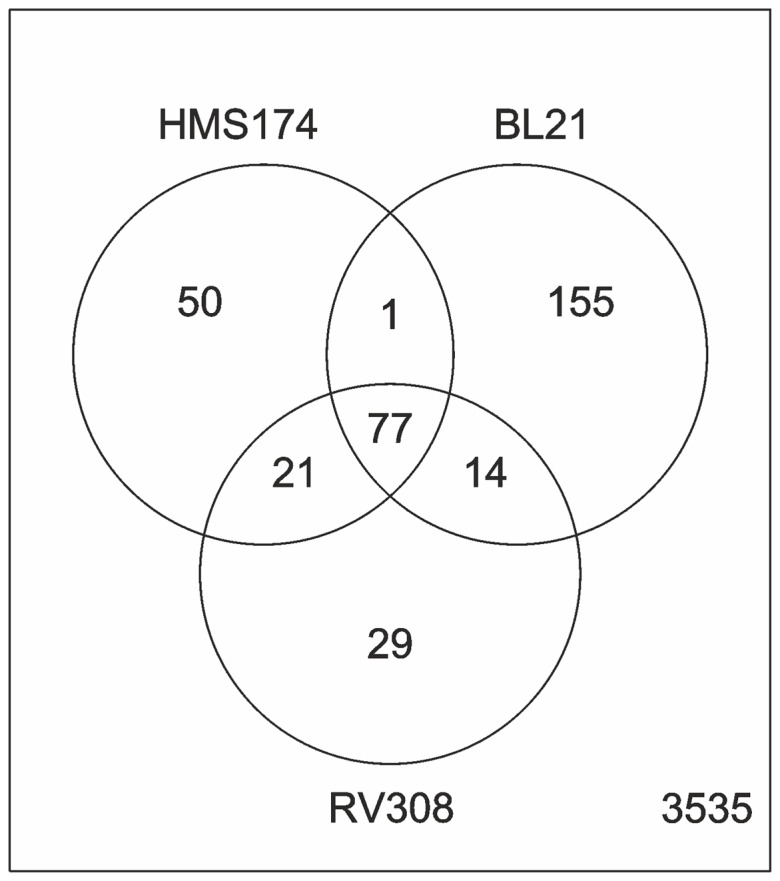
Venn diagram. The venn diagram shows a summary of the log-fold changes of batch transcription data, which revealed 347 genes as differentially expressed in all three strains. Overlaps represent differentially expressed genes that belong to two or all three data sets and not taking into account the direction of regulation (up or down). 3535 genes did not fulfill the filtering criteria of a log-fold change of one and were excluded from the microarray data analysis.

In a first step of data analysis, log-fold changes were compared among the three strains. For this purpose, 3882 genes available on both the BL21 and the K–12 array (further named as commonset) were analyzed. The Venn diagram (Figure 3) provides a visual summary of the log-fold changes for all 3882 genes in the different strains. It showed that 347 genes were differentially expressed (absolute log-fold change greater than one and p-value smaller than 0.05) in at least one strain whereas 3535 genes were not. Fifty genes were differentially expressed in HMS174, 29 in RV308, and 155 in BL21. Seventy-seven genes were differentially expressed in all three strains. These numbers confirm a closer resemblance of the transcription profiles of the two K–12 strains in contrast to the B strain where the greatest portion of differentially expressed genes was found.

#### Functional characterization of differentially expressed genes

For knowledge-based functional mapping of the differentially expressed genes, classification and annotation of genes into functional groups were accomplished using gene ontology (GO) terms [36]. For *E. coli* systems, the GO classification for biological processes (GOBP), molecular function (GOMF), and cellular components (GOCC) was sourced from the GenProtEC (*E. coli* genome and protein database, http://genprotec.mbl.edu/ [37]); the classification system for the cellular and physiological roles of *E. coli* gene products was sourced from the RegulonDB (*Escherichia coli* K–12 transcriptional network, http://regulondb.ccg.unam.mx/ [38]), providing information about operons and regulatory networks used.

Functional characterization of all 347 differentially expressed genes as shown in the Venn diagram (Figure 3) using GOMF yielded the following as the top three GO terms: GO:0005515 (protein binding, 56 genes), GO:0016740 (transferase activity, 42 genes), and GO:0003824 (catalytic activity, 30 genes). Applying the same procedure for GOBP yielded GO:0006810 (transport, 74 genes), GO:0042330 (taxis, 31 genes), and GO:0006355 (regulation of transcription, DNA dependent, 26 genes). For the GenProtEC classification, the terms “membrane” (90 genes), “cytoplasm” (71 genes), and “inner membrane” (63 genes) emerged as the most abundant terms, and “motility” (32 genes) claimed a notable part of the data set.

This functional grouping gives an overall view of the differentially expressed genes but still does not specify characteristic information about a particular host. To obtain more specific information, we examined the genes that were differentially expressed only in a single strain, using the GenProtEC classification system. Terms unique for an individual host were investigated in more detail to reveal specific properties of the strains (Table 4).

**Table 4:** GenProtEC classification.

host	multifunctional term	number	genes
**HMS174 (50 genes)**	Carbohydrates/Carbon compounds	1.1.1	7
	Anaerobic respiration	1.3.7	3
	Peptidoglycan (murein)	1.6.7	3
	Repressor	3.1.2.3	3
	The PTS Mannose-Fructose-Sorbose (Man) Family	4.4.A.1	3
**RV308 (29 genes)**	Sulfur metabolism	1.8.2	4
	Motility (incl. chemotaxis, energytaxis, aerotaxis, redoxtaxis)	5.3	3
	Glutamate/aspartate	4.S.75	3
**BL21 (155 genes)**	Fe-acquisition	5.5.7	11
	Prophage genes and phage related function	8.1	9
	Outer membrane	7.4	8

Unique terms for the different strains in the GenProtEC cell function assignment. For HMS174, 50 unique differentially expressed genes were used for the classification system; for RV308, there were 29 unique differentially expressed genes; and for BL21, 155 genes.

#### Detailed transcription analysis of selected groups in view of host performance

The achievement of high yields and growth rates is essential for optimal protein production in a bioreactor. Such cultivation conditions normally differ from actual environmental conditions, and important characteristics for optimal cell growth in nature can be obsolete for industrial applications. To identify futile as well as high-energy–consuming cellular processes as possible modification targets, we performed detailed host analysis. Consequently, this study was focused on selected differentially expressed pathways and groups (Table 4). The GO term “iron (Fe) acquisition” was detected as differentially expressed among the strains and was therefore investigated more closely. The entire “motility” was detected as differentially expressed among the hosts and thereby closer evaluated. Furthermore, “sugar transport” for enabling optimal growth and as a fundamental cultivation attribute of *E. coli* “acetate-related genes” was analyzed in more detail.

- *Iron (Fe) acquisition*

**Figure 4:**
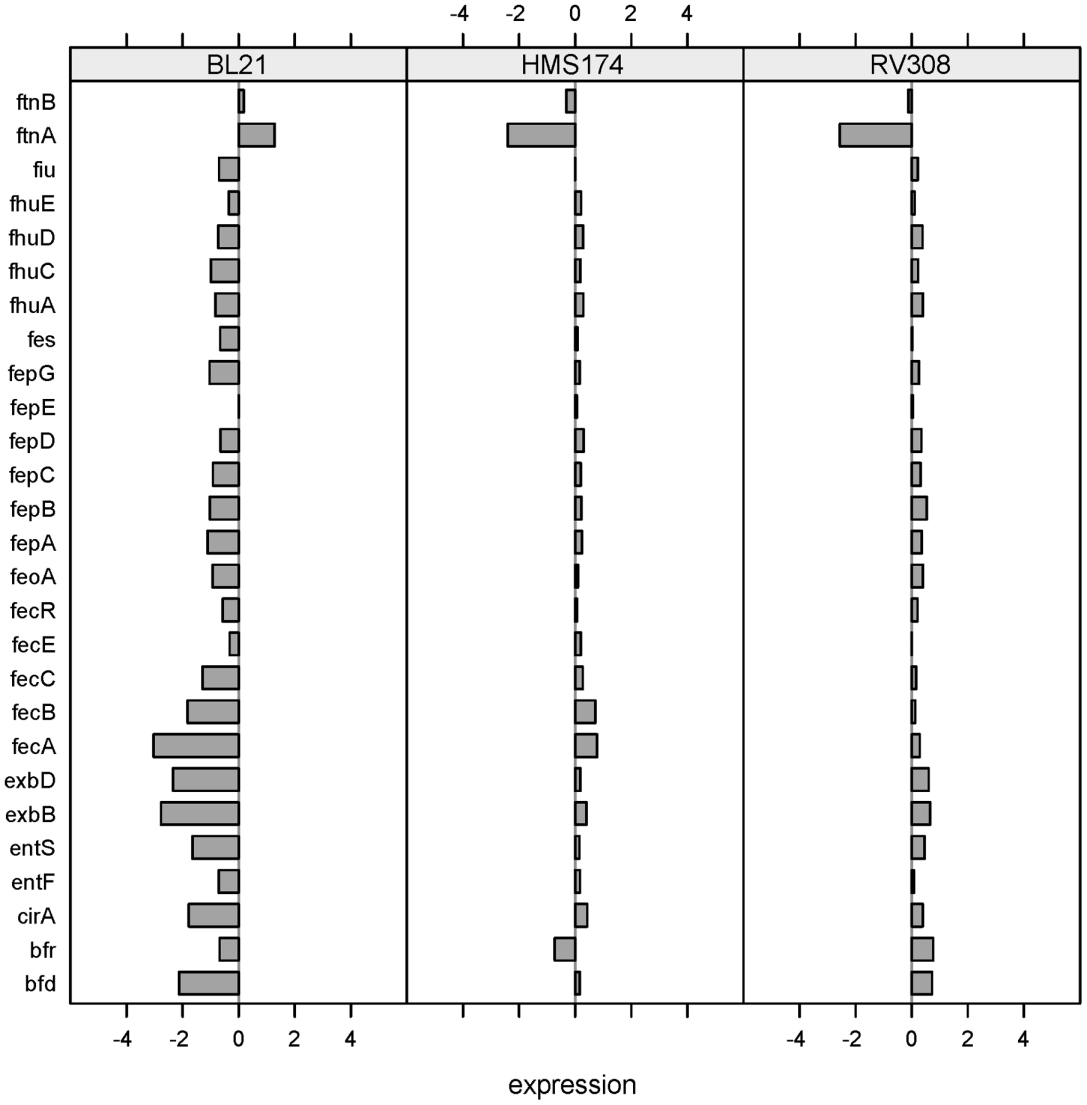
Differential responses: iron acquisition. Log-fold changes of all three strains for the GenProtEC term “iron acquisition.”

A high number of differentially expressed genes was detected in the group “Fe acquisition” and consequently selected for closer investigation. In BL21, 33% of the genes assigned to the term “5.5.7 – Fe acquisition” showed an absolute log-fold change greater than one (Figure 4).

This group is regulated by the global regulator and transcriptional activator for “**F**erric **U**ptake **R**egulation” (FUR). Genetic repression occurs when iron (Fe^2+^) binds to FUR [39], and activation is triggered by the cAMP–CRP complex. Of the analyzed commonset, FUR has a known influence on 75 genes [38]. Sixty-three of these genes showed a lower expression level than the reference in the BL21 data set, and of these, 15 genes even had a log-fold change smaller than (−1). Only 19 genes in RV308 and 17 genes in HMS174 showed a down-regulation in comparison to the reference. Because the mRNA expression levels of *fur*, the global regulator and activator, showed no significant differences among the three investigated hosts, a higher protein activity in the BL21 strain is assumed to counterbalance the down-regulation of FUR-repressed genes in BL21.A mutation in the cAMP–CRP complex binding site of *fur* and two silent point mutations in the *fur* coding sequence of BL21 (AM946981) were identified by sequence comparison in contrast to the available K–12 sequence information of MG1655 (U00096). However, the consequences of the mutations in terms of influencing binding affinity and mRNA stability are not well understood.

- *Motility*

Motility allows cells to move spontaneously towards a nutrient gradient through rotating and tumbling but is also a high-energy–demanding process. Because shut down of motility was indicated in two hosts, we investigated the flagellar machinery on transcriptional level.

**Figure 5:**
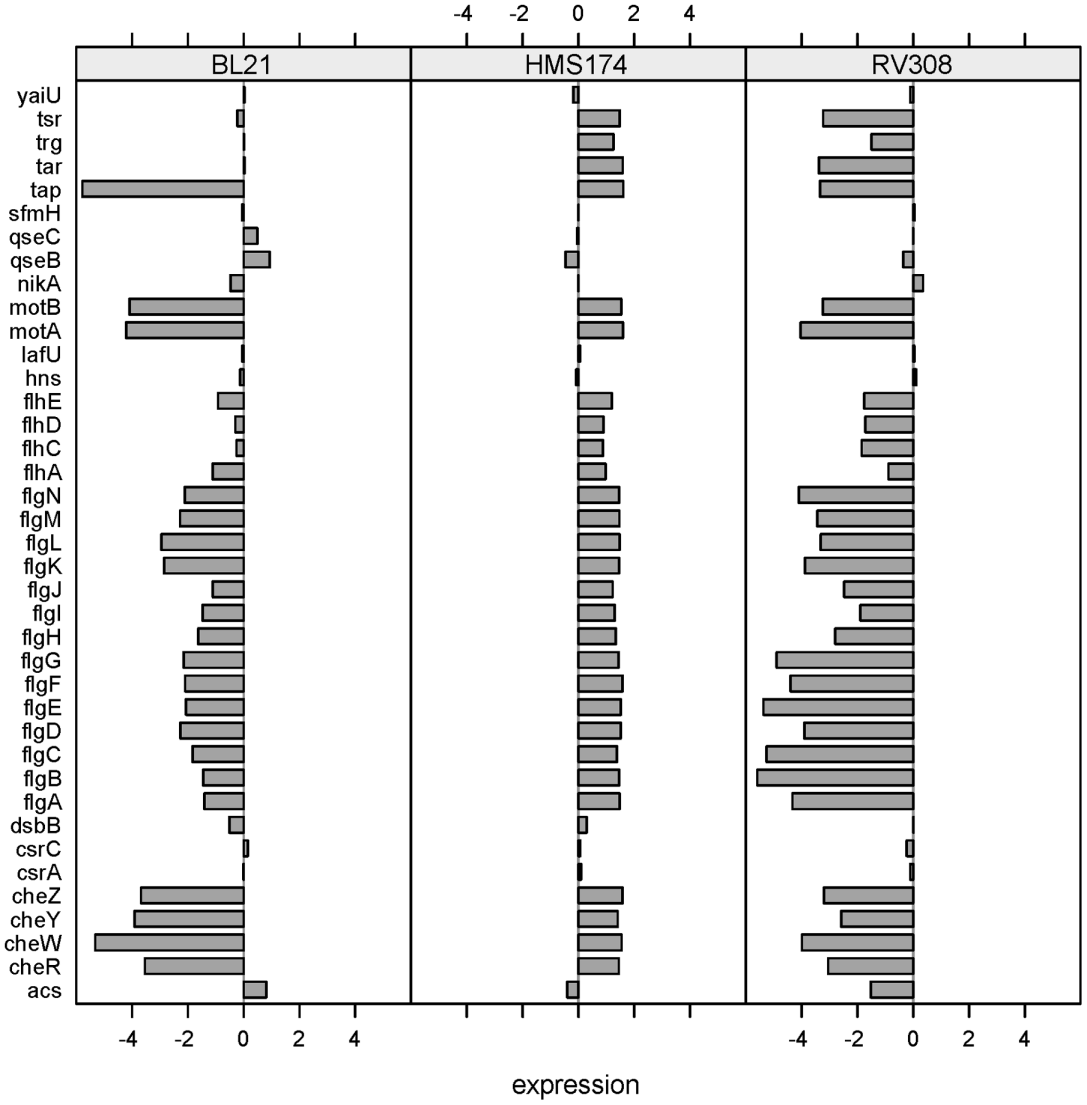
Differential responses: motility. Log-fold changes of all three hosts for the GenProtEC term “motility.”

RV308 as well as BL21 showed very low mRNA levels for the GenProtEC term “5.3 – motility” in contrast to the K–12 strain HMS174. Up-regulation of genes involved in motility was detected only for *E. coli* HMS174 (Figure 5). This finding suggests that the entire group is inactive or dysfunctional in BL21 and RV308.

**Figure 6:**
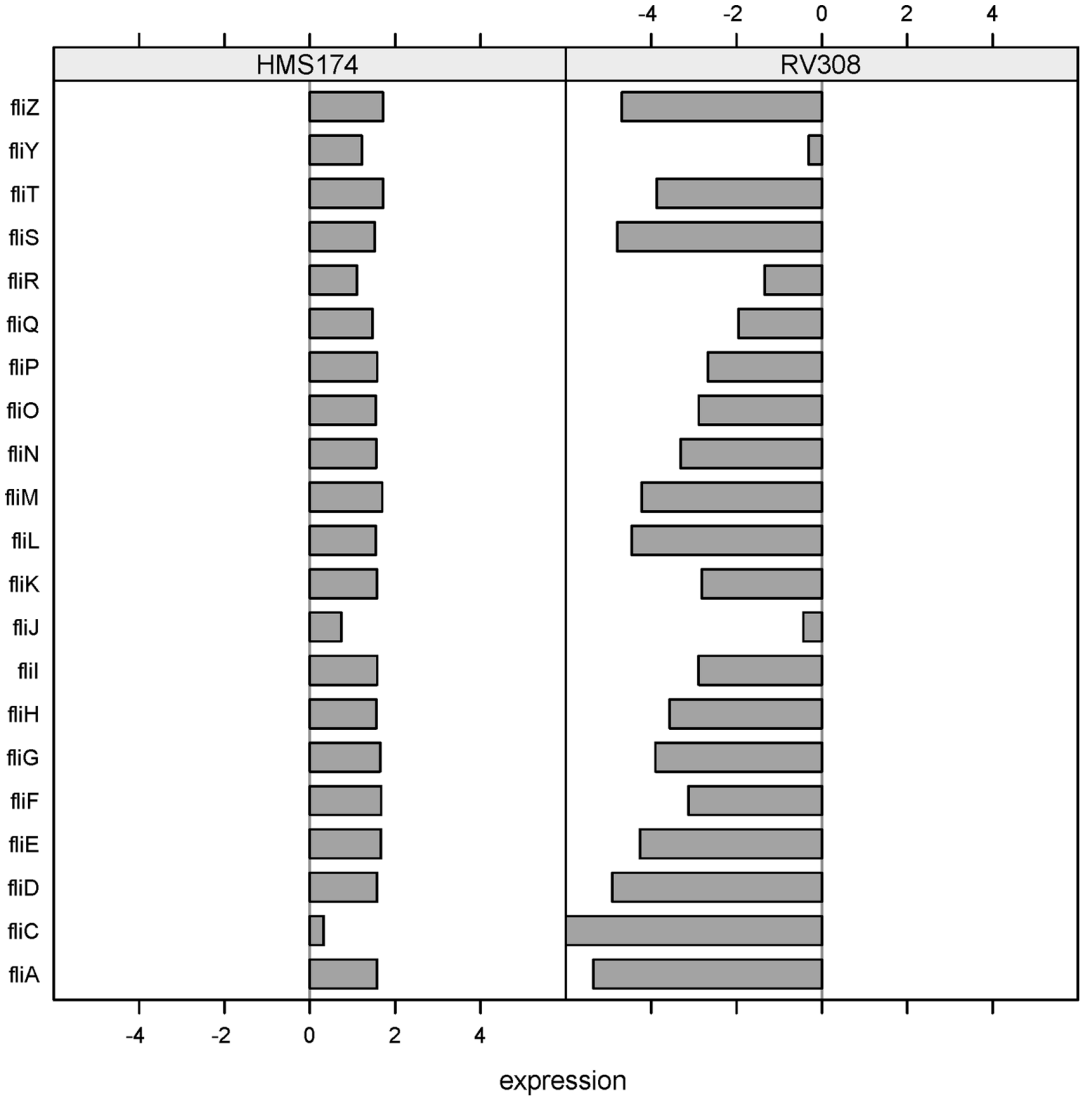
Differential responses: *fli*-operon of K–12 strains. Log-fold changes of HMS174 and RV308 for all genes of the *fli*-operon. These genes are absent on the BL21 genome because of an insertion element and consequently not in the commonset. Therefore, no BL21 mRNA expression data were available.

In the BL21 strain, the deletion of the *fli*-operon because of an insertion element can be assigned to this phenomenon [11]. For RV308, no such sequence information has been available up to now, but the microarray expression data showed a strong down-regulation of the whole *fli*-operon (Figure 6). Laboratory strains of *E. coli* W3110 have lost motility because of the loss of the FliA sigma factor 28 (σ^28^) during passage and storage [40]. This sigma factor controls the transcription of many of the flagellar and chemotaxis genes [41]. Lack of the sigma factor and the consequent loss of motility are irrelevant for stirred liquid cultures, as is the scenario in common industrial bioprocesses. The strain gains growth benefit and improved cell fitness through robustness and higher growth rates which has been approved for lysogeny broth liquid cultures [42]. The presence of the *fliA* gene on the RV308 genome was confirmed by PCR (data not shown). Hence, the non-expression of the flagellar genes cannot be assigned to the absence of the sigma factor.

**Figure 7:**
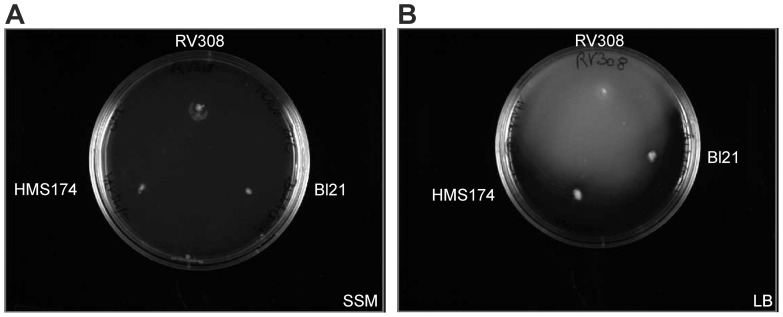
Motility test of the three hosts. Semi-synthetic agar plate (A, glucose present) with colonies of RV308 (top), HMS174 (bottom left), and BL21 (bottom right). None of the three strains showed swarming on the plate. Lysogeny broth agar plate (B, no glucose present) with colonies of RV308 (top), HMS174 (bottom left), and BL21 (bottom right). Only RV308 showed motile behavior by swarming on the plate.

Additionally, strains were tested for swarming motility on agar plates, which showed that the flagellar apparatus was induced and functional in RV308 and non-induced or dysfunctional in the other two strains (Figure 7). Usually, glucose inhibits transcription of the *flhD* operon [41], preventing the formation of the flagellar apparatus, and no swarming motility occurs, as observed for RV308. Therefore, the non-expression of the flagellar genes in RV308 during the batch cultivations can be explained by the suppression of motility with the presence of glucose in the media. HMS174 showed no motile behavior on the plates. During the batch cultivations, the gene expression levels of flagellar genes were up-regulated, but this gave no information about the functionality of the flagellum itself. However, the regulation of the HMS174 flagellar apparatus seemed to be defective because there was expression of flagellar genes during usually non-inducing conditions (glucose excess in batch mode). BL21 results confirmed an already known dysfunctional motile apparatus [43] by exhibiting no swarming on the plates as was observed during the cultivations.

In this study, both *E. coli* BL21 and RV308 showed higher specific growth rates than HMS174. According to Fontaine et al., the loss of motility is likely to result in a greater absolute availability of RNA polymerase and consequently free energy for other processes within the cell [42]. The released resources were presumably used for the growth acceleration that was observed for both hosts. Consequently, the strains RV308 and BL21 gained a growth advantage over HMS174 because of the non-expression of the flagellar genes (Figure 5, Figure 6).

- *Glucose transport and acetate metabolism*

Because optimal transport of carbohydrates/metabolites and acetate metabolism are fundamental attributes of *E. coli* cultivations and important characteristics for a production host, we focused especially on this group of genes.

**Figure 8:**
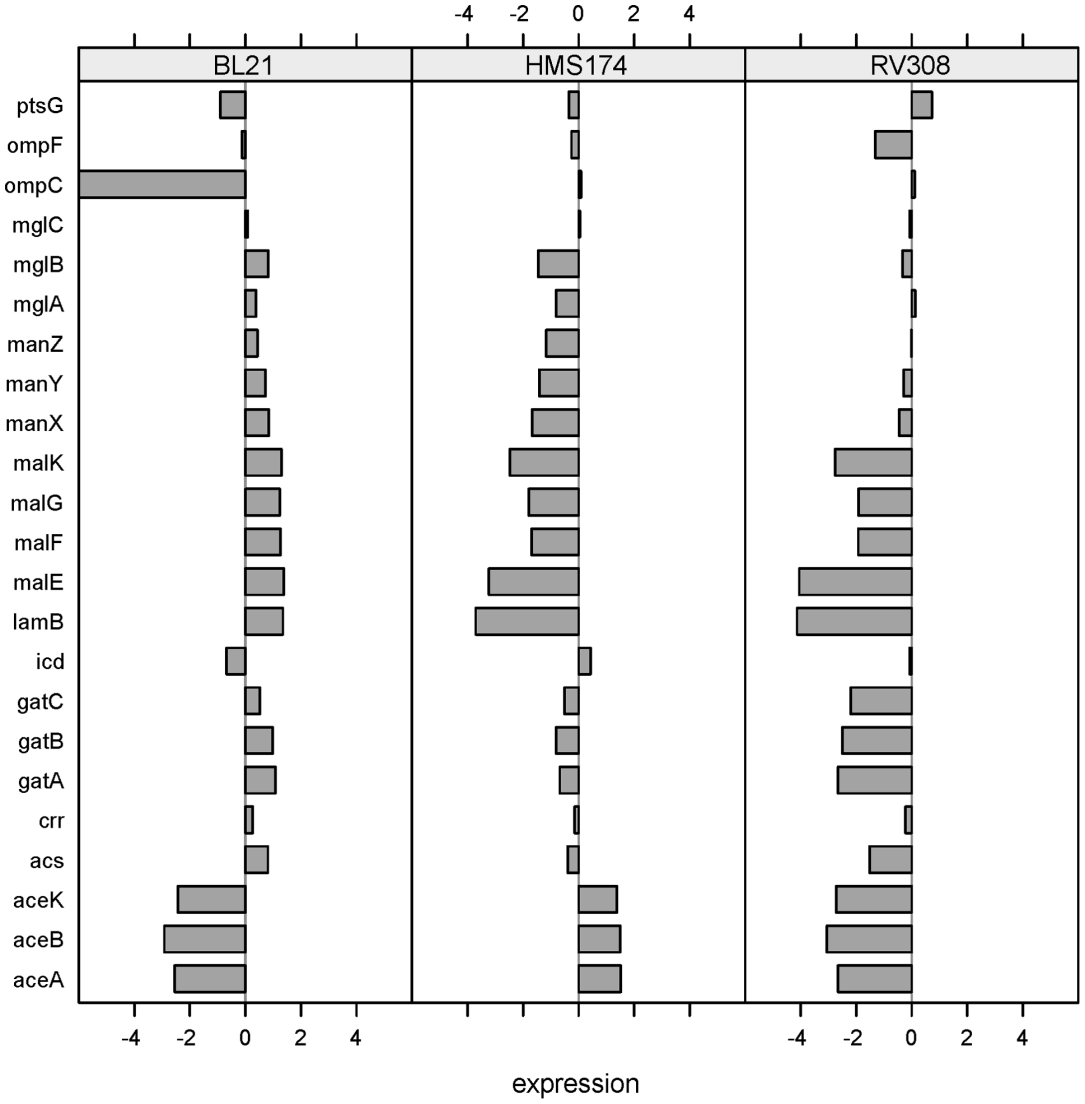
Differential responses: Glucose transport and acetate metabolism. Log-fold changes of all three strains for selected genes involved in carbon use, the TCA cycle, glyoxylate shunt, and acetate catabolism.

The functional superfamily of the phosphoenolpyruvate-dependent sugar transporting phosphotransferase system (PTS) is involved in the transport and phosphorylation of a large number of carbohydrates, in acetate formation [44], in chemotaxis, and in the regulation of some metabolic pathways [26]. In the PTS, differentially expressed genes were detected among the three hosts. GatABC, the galactitol PTS permease, and ManXYZ, the mannose PTS permease, showed higher expression levels in BL21 than in the K–12 strains (Figure 8). Because both are under positive control by cAMP–CRP [38], the higher transporter activity of BL21 can be attributed to the elevated cAMP level (Table 3). PtsG/Crr, the glucose-specific PTS permease, is also under positive control by cAMP–CRP [38] but did not show higher mRNA levels in one of the hosts (Figure 8).

In terms of carbon transport, the membrane permeability also had to be considered. Additionally, for BL21, the GenProtEC term “outer membrane” was found as differentially expressed only in this strain (Table 4). The gene *ompC*, encoding the outer membrane porin C, is dysfunctional in BL21 because of an insertion element. Therefore, the membrane composition of BL21 differs from that of the K–12 strains because OmpC has to be replaced by other cellular components. No differential expression was measured among the strains for the second outer membrane porin *ompF* (Figure 8). Ferenci et al. stated that during glucose-limited conditions, the LamB porin enables facilitated entry of glucose into the cell [45]. Higher *lamB* gene expression values of the B strain support a higher activity in BL21, even under non-limiting glucose conditions during batch cultivations. Therefore, it is assumed that BL21 counterbalances the missing outer membrane porin by induction of *lamB* to improve glucose transport. Additionally, we detected changes in the ATP-binding cassette (ABC) transport system in the inner membrane of the investigated hosts. In particular, the expression levels of genes for the maltose ABC transporter *malKFGE* and for the galactose ABC transporter *mglABC* were higher in BL21 (Figure 8). The induction of the galactose ABC transport system has also been observed in glucose-limited cultures for optimizing carbon transport [45]. Presumably, different substrate transport mechanisms and higher cAMP levels (Table 3) enable higher growth rates of the BL21 strain, as observed during the cultivations (Figure 2A).

In the context of acetate metabolism, the investigated K and B strains showed different acetate accumulation (Table 1). Noronha et al. reported that the higher flux through the tricarboxylic acid cycle (TCA) and a more active glyoxylate shunt (encoded by *aceBAK*) in the B strain compared to the K–12 strain JM109 are responsible for lower acetate levels [46]. In this study, the expression levels of *aceBAK* of the K–12 strains were higher compared to BL21 (Figure 8) and no higher activity could be detected in BL21. Moreover, the mRNA expression level of *icd*, encoding isocitrate dehydrogenase, was lower in BL21, as well (Figure 8). This protein enables *E. coli* to perform a rapid shift between TCA and glyoxylate bypass [47]. The gene expression levels of further acetate-related genes were not remarkably elevated. Exclusively, the gene *acs* encoding the acetyl-CoA synthetase was detected at higher levels in the B strain (Figure 8). Acetyl-CoA synthetase activates the cell's ability to convert acetate to acetyl-CoA. Additionally, high cAMP levels induce transcription of *acs* [48], which was detected in this study as elevated for the B strain (Table 3). Altogether, the higher *acs* level appears to have strongly contributed to the lower acetate accumulation in BL21 in contrast to the K–12 strains.

### Proteome analysis

Protein expression cannot be derived from transcriptome data alone; to obtain a more complete picture of regulation, both levels must be investigated. For this purpose, we applied 2D-DIGE and protein identification of selected peptides by MS/MS. Proteins were regarded as significantly altered if they exhibited at least a 2.0-fold increased or decreased abundance and a corresponding Student's t-test of <0.01 (Table 5). Most of the altered proteins were found in the pH range of 4 to 7 because the majority of *E. coli* proteins are acidic. Because of the similarity of the K–12 strains, only 42 proteins were differentially expressed in contrast to the comparison with *E. coli* BL21 (179 differentially expressed vs. HMS174 and 194 differentially expressed vs. RV308; Table 5).

**Table 5:** Altered proteins.

Comparison	Acidic range (pH 4–7)	Basic range (pH 6–11)	Total range (pH 4–11)
BL21 vs. HMS174	161	18	179
BL21 vs. RV308	176	18	194
RV308 vs. HMS174	36	6	42

Proteins detected as significantly altered in the acidic, basic, and total ranges of the 2D-DIGE gels (at least 2.0 fold increased or decreased abundance and a corresponding Student's t-test <0.01).

**Figure 9:**
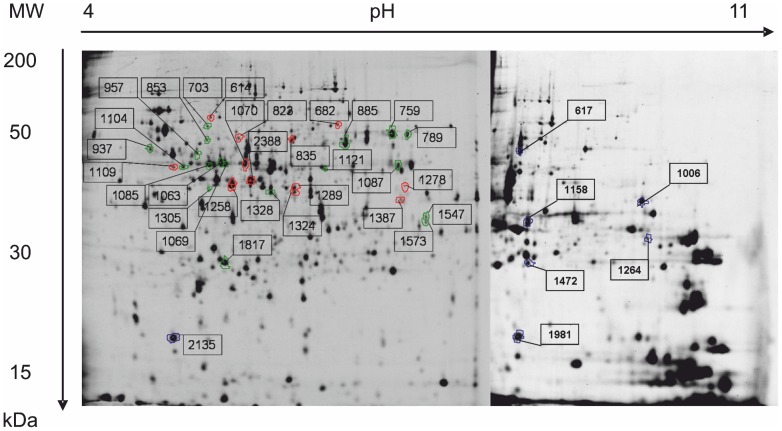
2D-gel map. The 2D-gel map of cellular proteins of the *E. coli* mixture separated on 4–7 and 6–11 immobilized pH gradient (IPG) strips followed by 12.5% w/v sodium dodecyl sulfate polyacrylamide gel electrophoresis (SDS-PAGE) and visualized by fluorescence labeling. Identified proteins are marked with numbers and listed in Table 6.

Out of 245 altered proteins, 50 spots were selected for analysis by mass spectrometry, and 38 were identified (Figure 9). Table 6 lists these proteins and includes all protein and corresponding gene expression ratios as well as the MS/MS data. Most of the proteins showed comparable expression at the proteome and transcriptome level (e.g. GatD, AceA, and MalE), but divergent levels also were observed (e.g. CysP and ProS).

**Table 6:** Comparison of proteome and transcriptome data.

Protein	Spot number	MW (Da)	pI	Coverage (%)	MS^2^ data (peptides)	PROTEIN EXPRESSION VALUE			Gene	GENE EXPRESSION VALUE		
						BL21-HMS174	BL21- RV308	HMS174-RV308		BL21-HMS174	BL21- RV308	HMS174-RV308
**AceA**	1069	47777	5.16	20	5	−6.24	−1.05	5.95	***aceA***	−3.96	0.08	4.04
**AceA** **(+Icd)**	1085	47777	5.16	15	3	−18.28	−15.35	1.19				
**AldA**	853	52411	5.07	50	5	−8.66	−5.07	1.71	***aldA***	−1.28	−0.74	0.53
**AroF**	1289	39065	5.42	60	5	9.95	16.76	1.68	***aroF***	2.46	1.60	−0.85
**BtuB**	703	68372	5.36	17	4	−27.75	−36.10	−1.30	***btuB***	−4.30	−4.36	−0.06
**CysP**	1158	37591	7.78	45	4	15.60	1.08	−2.14	***cysP***	0.97	2.30	1.33
**DppA**	885	60483	6.21	42	3	−2.83	−3.30	−1.16	***dppA***	−1.68	−1.82	−0.15
**FliD**	937	48427	4.28	40	5	−16.72	1.49	24.97	***fliD***	NA	NA	7.83
**FliY**	1817	29021	6.21	57	5	−57.19	−29.51	1.94	***fliY***	NA	NA	1.54
**GapA**	1387	35681	6.61	39	4	2.83	4.80	1.70	***gapA***	0.17	0.11	−0.05
**GatD**	1278	37822	5.94	37	4	9.69	11.44	1.18	***gatD***	3.56	4.09	0.53
**GdhA**	1087	48778	5.98	49	5	−2.81	−2.98	−1.06	***gdhA***	−1.75	−1.95	−0.19
**GuaB**	789	52275	6.02	62	5	−2.56	−2.04	1.26	***guaB***	−1.40	−1.17	0.23
**HisD**	957	46481	5.06	42	4	−3.85	−9.17	−2.38	***hisD***	0.49	−1.09	−1.58
**Icd**	1063	46070	5.15	55	7	−25.46	−15.52	1.64	***icd***	−1.06	−0.73	0.34
**Icd**	1070	46070	5.15	61	6	5.80	11.38	1.96				
**Icd** **(+AceA)**	1085	46070	5.15	22	4	−18.27	−15.34	1.19				
**IlvD**	682	66174	5.59	50	6	8.03	6.57	−1.22	***ilvD***	1.23	1.18	−0.05
**IlvG**	823	59760	5.22	18	4	4.23	4.73	1.12	***ilvG***	−0.81	−0.83	−0.01
**LeuA**	835	57604	5.47	45	6	2.62	3.17	1.21	***leuA***	−0.21	0.26	0.47
**LivJ**	2388	39223	5.54	35	5	−1.42	2.53	3.59	***livJ***	−0.07	0.73	0.80
**MalE**	1258	43360	5.53	56	5	9.69	22.89	2.36	***malE***	4.44	5.27	0.83
**OppA**	759	60975	6.05	48	5	−43.13	−29.17	1.48	***oppA***	−1.39	−1.11	0.27
**PepB**	1121	46436	5.6	34	4	−4.44	−4.43	1.00	***pepB***	−0.36	−0.43	−0.07
**Pgl**	1305	36570	5.06	32	4	−9.40	−10.18	−1.08	***pgl***	NA	NA	0.19
**ProS**	614	63640	5.08	56	5	2.97	2.35	−1.26	***proS***	0.00	−0.49	−0.50
**PyrB**	1547	34463	6.12	54	5	−26.80	−1.99	13.50	***pyrB***	−3.90	−0.76	3.14
**PyrB**	1573	34463	6.12	54	5	−26.07	−1.43	18.23				
**PyrI**	1981	17338	6.89	58	6	−18.58	1.03	18.05	***pyrI***	−3.90	−0.59	3.31
**RcsB**	1472	23656	6.85	32	3	−12.76	−7.36	1.73	***rcsB***	NA	NA	0.43
**Rho**	617	47032	6.75	43	9	36.26	18.10	−2.00	***rho***	1.16	1.33	0.17
**SerC**	1324	39986	5.37	58	5	5.88	9.57	1.63	***serC***	0.34	0.99	0.66
**SerC**	1328	39986	5.37	43	5	−7.54	−4.57	1.65				
**Tpx**	2135	17995	4.75	58	5	−2.28	−1.27	1.80	***tpx***	0.32	0.51	0.19
**YcgR**	1264	27897	8.99	26	2	−9.92	1.27	12.63	***ycgR***	−6.61	−1.78	4.84
**YchF**	1104	39984	4.87	35	4	−7.09	−8.39	−1.18	***ychF***	0.65	−0.12	−0.77
**YchF**	1109	39984	4.87	33	5	3.91	5.02	1.28				
**YncE**	1006	38589	9.22	32	4	−38.00	−33.75	1.13	***yncE***	−3.32	−3.48	−0.16

Protein and corresponding gene expression ratios of all identified proteins including the MS/MS data.

#### Abundance of proteins related to transport, motility, iron acquisition, and acetate

MalE, the periplasmic maltose-binding protein and member of the maltose transport system, and GatD, the galactitol-1-phosphate dehydrogenase, confirmed a higher carbon transport activity in BL21 batch cultivations as was also found at the transcriptome level (Table 6).

YcgR, involved in flagellar motility, was under-expressed in BL21 and RV308 in contrast to HMS174. Equivalent results were gained from the microarray data. FliD and FliY, two more proteins of the flagellar machinery, were also found in the proteome of *E. coli* HMS174, but only very low expression levels were obtained for the other two strains, which can be considered as non-expression of these proteins (Table 6). Thus, the diverse behavior in chemotaxis and motility for HMS174 detected at the transcriptome level was demonstrated at the proteome level, as well.

For the GenProtEC term “Fe acquisition,” no differentially expressed protein was identified. AceA, the second enzyme of the glyoxylate bypass, was under-expressed in BL21 in contrast to both K–12 strains, similar to the pattern from the transcription analysis. Therefore, the higher glyoxylate shunt activity as proposed in literature [46] could not be confirmed at the proteome level.

#### Further differentially expressed proteins

Proteins involved in amino acid biosynthesis pathways like HisD, IlvD, IlvG, LeuA, LivJ, ProS, or SerC were differentially expressed based on proteome patterns, in contrast to the transcriptome analysis in which no significant alterations of these genes were detected. In BL21, IlvD, IlvG, LeuA, and ProS showed higher expression, in contrast to the K–12 strains, which were involved in the biosynthetic process of leucine, isoleucine, and valine. HisD, involved in the histidine biosynthetic process, was detected at higher expression levels in both K–12 strains, and for LivJ (involved in the valine and leucine biosynthetic process); the highest expression levels were found for HMS174. Contrasting results were found for SerC (involved in the serine biosynthetic process), which was detected twice, once at higher expression levels for the B strain and once for the K–12 strains.

Additionally, AroF (involved in the aromatic amino acid biosynthetic process), GdhA (the glutamate biosynthetic process), and PyrB and PyrI (which encode the aspartate transcarbamylase) were detected as differentially expressed at the proteome and transcriptome levels, and expression results were comparable between the molecular levels (Table 6).

BtuB (an outer membrane receptor for transport of vitamin B12) was unexpressed in BL21 at the protein level and further identified to contain an internal stop codon in the mRNA coding sequence. Therefore, another member of the outer membrane porins, like *ompC*, is dysfunctional in BL21.

The detected variations of the activity of the individual amino acid pathways among the hosts are highly relevant in terms of recombinant gene expression. Therefore, the highest similarity of the amino acid composition of the applied host and the target protein will be one of the key criteria of host selection.

Some identified proteins (AceA, Icd, SerC, and PyrB) were present as different isoforms on the gels and thus identified in neighboring spots (Figure 9). This finding necessitates a more complex analysis of proteome patterns. Possible explanations for the detected isoforms of the same protein are different post-transcriptional or post-translational modifications (e.g. sulfation, acylation, etc.), or single nucleotide polymorphisms of the diverse *E. coli* strains.

## Conclusions

Distinct recombinant systems reveal great differences in growth behavior, product yields, and other cultivation characteristics, such as different accumulation of by-products or specific growth rates. Accessorily, the effect of a particular host on recombinant protein production and vice versa is still not well understood. Even gene and protein expression patterns of related subspecies can vary considerably, as this study has confirmed. Therefore, we investigated the global strain characteristics and transcriptional and translational patterns of three *E. coli* B and K strains to extend the specific host knowledge and identify possible modification targets. Overall, this approach provides the basis for directed optimization of microbial cell factories.

Cultivation characteristics revealed great differences among the two K–12 strains and one B strain as the B strain achieved higher growth rates, higher biomass yields, and lower acetate accumulation. However, the K–12 strain RV308 showed great potential with its very high specific growth rate in the later batch phase and a very high biomass per substrate conversion rate. Due to the high concentrations of acetate formed during all cultivations the applied batch strategy is not applicable for production processes of valuable molecules as the conditions can be unfavorable for the host and/or product expressed. Thereby, other strategies should be used where acetate formation is avoided like e. g. the fed-batch mode.

The results from the in-depth analysis of transcriptome and proteome data suggested the improvement of the glucose transport processes of the K–12 strains and a shutdown of non-essential pathways like the flagellar machinery for strain engineering. In this way, host performance can be enhanced by increased exploitation of the entire cellular machinery, resulting in higher system productivity of K–12 strains. Overall, without host engineering or advanced process strategies, BL21 would be the candidate of choice for processes requiring fast growth rates, high biomass yields, and low accumulation of by-products.

The information presented here elucidates differences of strain behavior and cultivation performance. In order to discriminate between genotypic and phenotypic triggered effects, which in turn enables directed host engineering, genome sequencing and annotation is intended. Altogether, this valuable in-depth host knowledge will help to further improve future recombinant system design and set-up.

## Supporting Information

File S1 **Nanostring data.** Transcript variant data for BL21, RV308 and HMS174 batch cultivations: NanoString code set details, Nanostring mRNA counts [experiment BL21, RV308 and HMS174], Nanostring log-fold changes in mRNA counts compared to the references sample (mixture of all samples) and DNA microarray data log-fold changes in mRNA counts compared to the reference sample (see FileS1.xlsx).
(XLSX)
